# On-site detection of MERS-CoV infections in a camel slaughterhouse in Kenya using a commercial rapid antigen test

**DOI:** 10.3389/fvets.2025.1675847

**Published:** 2025-09-15

**Authors:** Brian Maina Ogoti, Victor Riitho, Jordi Rodon, Nyamai Mutono, Julia Tesch, Julius Oyugi, Marianne W. Mureithi, Victor M. Corman, Christian Drosten, Johanna Wildemann, Samuel M. Thumbi, Marcel A. Müller

**Affiliations:** ^1^Department of Medical Microbiology and Immunology, University of Nairobi, Nairobi, Kenya; ^2^Institute of Tropical and Infectious Diseases, University of Nairobi, Nairobi, Kenya; ^3^Center for Epidemiological Modelling and Analysis, University of Nairobi, Nairobi, Kenya; ^4^The Blizard Institute, Queen Mary University of London, London, United Kingdom; ^5^Discovery Science International AIDS Vaccine Initiative Africa, Nairobi, Kenya; ^6^Institute of Virology, Charité–Universitätsmedizin Berlin, Freie Universität Berlin, Humboldt-Universität zu Berlin, and Berlin Institute of Health, Institute of Virology, Berlin, Germany; ^7^Paul G. Allen School for Global Health, Washington State University, Pullman, WA, United States; ^8^Institute of Clinical Research, KAVI, University of Nairobi, Nairobi, Kenya; ^9^German Centre for Infection Research, Berlin, Germany; ^10^Virus Diagnostics, LaborBerlin, Berlin, Germany; ^11^Faculty of Life Sciences, Humboldt Universität zu Berlin, Berlin, Germany; ^12^Institute of Immunology and Infection Research, University of Edinburgh, Edinburgh, United Kingdom

**Keywords:** middle east respiratory syndrome, coronavirus, rapid test, epidemiology, dromedary camel (*Camelus dromedarius*), Kenya, slaughterhouse, MERS

## Abstract

**Background:**

Middle East respiratory syndrome coronavirus (MERS-CoV) poses a significant public health risk, with dromedary camels being the primary reservoir hosts. Regular and systematic surveillance for MERS-CoV is limited by the lack of extensively validated, rapid, field-deployable diagnostic tools.

**Objective:**

We aimed to validate and implement a commercial MERS-CoV antigen test kit (Bionote, South Korea) for field surveillance of MERS-CoV in Kenya.

**Methods:**

We evaluated whether the Bionote MERS-CoV rapid antigen test can discriminate between two different MERS-CoV isolates representing clades A (EMC/2012) and C (Kenya/9954). We conducted an assay performance evaluation using 2,736 archived camel nasal swab samples with defined MERS-CoV RNA concentrations (10^3^–10^9^ MERS-CoV RNA copies/ml). Subsequently, we performed a prospective study at the central camel slaughterhouse in Isiolo, northern Kenya, testing 386 samples collected from March–April 2024.

**Results:**

MERS-CoV strain-specific testing showed consistent virus antigen detection for both applied MERS-CoV isolates, with no statistically significant differences in positivity thresholds. A receiver operating characteristic (ROC) curve analysis based on the 2,736 archived MERS-CoV clade C RNA-pretested camel samples identified a limit of detection (LOD) of 1.53 × 10^6^ RNA copies/ml. The estimated LOD at 90% probability (LOD_90_) was 5.01 × 10^5^ RNA copies/ml. Out of the 2,736 tested samples, 9 samples (0.33%) were positive in the MERS-CoV rapid antigen test showing a diagnostic sensitivity of 25% compared to RT-qPCR and a specificity of 100% (95% CI, 99.9–100%), with a Cohen’s Kappa of 0.40. Critically, the test demonstrated 100% sensitivity for infectious samples with viral loads >10^6^ copies/ml. All 9 samples had RNA genome copies/ml above the LOD. For 7/9 samples (78%) virus isolation was successful. In the prospective study, we identified 3/386 MERS-CoV-antigen positive camels by the rapid antigen test on-site which we confirmed by MERS-CoV upE- and orf1a-based RT-qPCR assays.

**Conclusion:**

The commercial Bionote MERS-CoV antigen test kit demonstrates reliable, clade-independent detection, enabling rapid MERS-CoV surveillance in camels in high-risk settings. The majority of antigen-positive samples contained infectious virus suggesting its applicability for assessing infection risks at slaughterhouses by the rapid test. The successful identification of MERS-CoV-infected camels at the point of slaughter underscores the critical importance of rapid diagnostics in high-exposure environments to mitigate zoonotic transmission and protect the health of slaughterhouse workers.

## Introduction

Middle East respiratory syndrome coronavirus (MERS-CoV) is a zoonotic threat with considerable pandemic potential. To date, more than 2,600 humans have been infected with more than 900 fatalities (1). MERS-CoV can cause severe respiratory distress in humans similar to COVID-19 (2), but subclinical infections appear to occur regularly (3, 4). Seroepidemiological studies suggest that camel handlers and workers might have the highest risk of contracting MERS-CoV (5–7). Camel slaughterhouses can be considered high-risk settings for zoonotic transmission due to extensive human-camel interaction during animal processing, aerosol production from slaughter activities, and crowded conditions in holding pens where camels await slaughter. Despite this, surveillance in slaughterhouses is constrained by logistical difficulties including limited cold chain infrastructure for sample transport and the limited application of field-deployable diagnostic tools. Therefore, rapid diagnostic tests can be critical for MERS-CoV surveillance and outbreak control, especially in remote high-risk settings.

Prototypical rapid diagnostic methods are often virus antigen tests that experience certain limitations such as low sensitivity especially when compared to highly sensitive quantitative PCR assays (8). However, the widely applied, easy-to-use SARS-CoV-2 antigen tests have proven valuable in identifying infectious individuals during the past pandemic. The typical LOD of a SARS-CoV-2 rapid antigen test is approximately 10^5^ to 10^6^ viral RNA copies/ml which coincided with the threshold of infectiousness especially during the ascending and peak phases of viral replication (9–11). Typically, SARS-CoV-2 genome copies/ml were 1,000- to 10,000-fold higher than infectious units/ml (12). In the case of MERS-CoV, we previously showed that the threshold for virus isolation success from early-phase clinical samples was above 10^5^ RNA copies/ml (13) suggesting that a rapid antigen test with an LOD in a range of 10^5^ to 10^6^ RNA copies/ml might be suitable to detect acute MERS-CoV infections in the peak phase.

The Bionote MERS-CoV antigen immunochromatographic test is one of the few commercially available rapid diagnostic platforms certified by the World Organisation for Animal Health (WOAH) (14). The lateral chromatographic flow assay is based on the detection of MERS-CoV nucleocapsid (N)-proteins via monoclonal antibodies. In a previous study, the rapid antigen test demonstrated 94% sensitivity and 100% specificity when validated against a MERS-CoV RT-qPCR using 571 camel nasal swabs (14). The initial validation study of the Bionote MERS-CoV antigen kit established basic performance metrics for the detection of Arabian MERS-CoV lineages showing an LOD of 10^5^ TCID_50_ per ml (14). To date, LOD determinations based on quantitative detection of viral genome copies by RT-qPCR have not been performed. Previous studies used the Bionote MERS-CoV rapid test under laboratory conditions (14). Laboratory environments do not fully recapitulate field conditions, which might suffer from reduced sensitivity due to inconsistent sample quality and dynamic environmental conditions (15). In addition, the currently available MERS-CoV antigen test was established for the detection of MERS-CoV EMC (clade A) and applied on MERS-CoV clade B-positive samples (14). Whether genetically distant MERS-CoV clade C strains, that are commonly circulating on the African continent, can be detected remains unknown.

To address these gaps, we aimed to (a) evaluate the detection consistency of the Bionote MERS-CoV antigen test of MERS-CoV clades A and C isolates, (b) develop comprehensive performance metrics and establish viral load thresholds using archived camel nasal samples from a previous MERS-CoV study in Kenya (7), and (c) evaluate the rapid antigen test performance in the field through a prospective MERS-CoV surveillance study at the Isiolo County camel slaughterhouse in Kenya.

## Methods

### Study design

#### Archived camel nasal samples

Archived camel nasal swab samples (*n* = 2,736) from our previous surveillance study in Kenya were re-tested using the Bionote MERS-CoV rapid antigen kit (example reactive swabs, Supplementary Figure 1). The methodology for the collection of these archived samples has been previously detailed (7). All archived camel nasal samples were previously analyzed using validated upE and orf1a RT-qPCR protocols (16, 17).

#### Prospective field study

A prospective cross-sectional field study was performed at a central camel slaughterhouse in Isiolo County, Kenya, from March to April 2024, with ethical approval granted by the Kenyatta National Hospital Ethics and Research Committee (protocol number P534/08/2020) and the Kenya National Commission of Science and Technology (NACOSTI, P/22/21987). The Isiolo County central camel slaughterhouse was strategically selected as the validation site based on our previous documentation of biphasic MERS-CoV incidence patterns in this location (7). As shown in our previous work, field studies on nomadic camels are hampered by limited infrastructure in remote regions, whereas abattoir hubs enable sustained daily testing. The camel slaughterhouse processes around 20 to 25 camels daily, with the slaughtered camels originating from pastoral communities across the northern region of Kenya. In the current study, a total of 386 post-mortem camel nasal samples were collected by a trained animal health technician from 8 to 12 dromedary camels per day, 5 days a week. Post-mortem swabbing was conducted on the caudal turbinate of the nose using Copan FLOQSwabs (Mast Diagnostica GmbH, Reinfeld, Germany), following a transverse incision above the nostrils to prevent contamination from the frontal nasal region.

A field testing unit was established within 1-km distance of the slaughterhouse to enable rapid sample transport in cool boxes and to perform the rapid antigen test within a few hours post sampling followed by real-time result reporting. The Bionote MERS-CoV antigen test kit was conducted according to the manufacturer’s instructions (see below), with positive samples subsequently confirmed by upE (16) and orf1a RT-qPCR (17).

### Laboratory procedures

#### MERS-CoV clade specific analysis and field testing in dromedary samples

Laboratory-cultured MERS-CoV strains representing clade A (accession: NC_019843.3) and clade C (accession: OR742171.1) were used for clade-specific validation of the Bionote MERS-CoV antigen test kit. The viruses were propagated in Vero E6 cells expressing TMPRSS2 (Vero E6-T, National Institute for Biological Standards and Control (NIBSC), 100978) maintained in Dulbecco’s modified Eagle’s medium supplemented with 10% fetal bovine serum (FBS) at 37°C in 5% CO₂. To establish the LOD of the rapid antigen kit, each MERS-CoV virus strain was prepared in 10-fold serial dilutions ranging from 10^6^ to 10^1^ TCID_50_/ml (18, 19), and also quantified via upE RT-qPCR to determine the viral RNA copies/ml (16). For validation of the rapid antigen test with field camel nasal samples, virus isolation was attempted on archived MERS-CoV RT-qPCR-positive samples above 10^4^ RNA copies/ml, regardless of the result of the rapid antigen test, using Caco-2 cells. Archived nasal samples were inoculated onto 24-well plates with confluent Caco-2 cell monolayers cultured in antibiotic-supplemented DMEM containing 10% FBS and incubated at 37°C in 5% CO₂ for 72 h with daily monitoring for cytopathic effects (CPE). Successful viral isolation was confirmed by the presence of characteristic CPE and subsequent upE (16) and orf1a RT-qPCR assays (17).

#### Bionote MERS-CoV antigen test kit

The Bionote MERS-CoV antigen test kit (Bionote Inc., Hwaseong-si, South Korea) detects MERS-CoV N protein using highly specific monoclonal antibodies against a clade A strain as previously detailed (14). Test strips were maintained at ambient temperature and used in accordance with the manufacturer’s instructions. In summary, 100 μl of the camel nasal swab sample in universal transport medium was combined with 100 μl of assay diluent, and the test strips were evaluated after 15 min. Tests were considered positive when both the test and control lines were visible, negative when only the control line was present, and invalid when the control line was absent. To ensure compliance with WOAH validation requirements (20), all Bionote MERS-CoV antigen test readings were verified by two independent technicians.

#### Infectious virus titrations

Infectious MERS-CoV samples were titrated on Vero E6-T. Ten-fold dilutions of the MERS-CoV samples were transferred to Vero E6-T monolayers and incubated at 37°C and 5% CO_2_. Cells were inspected daily for the presence of virus-induced cytopathic effect under an inverted light microscope. After 5 days, titers were calculated by quantifying the dilution that caused 50% CPE in Vero E6-T cultures (TCID_50_/ml), according to the Reed and Muench method (19).

#### MERS-CoV RT-qPCR testing

RNA extraction from all Bionote antigen test-positive camel nasal samples identified during the prospective surveillance was performed using the MagnaPure 96-well plate nucleic acid extraction system (Roche, Penzberg, Germany) following the manufacturer’s protocol. MERS-CoV RNA detection and quantification was performed using the established upE RT-qPCR assay as the primary confirmatory method (16), with all positive samples undergoing additional confirmation using the MERS-CoV orf1a RT-qPCR, as previously described (17). Viral RNA concentrations were determined using a standard curve generated from serial dilutions of quantified MERS-CoV RNA standards, with viral loads calculated and expressed as RNA copies/ml. The diagnostic LODs of the upE and orf1a assays are 3.4 and 4.1 genome copies/reaction (16, 17).

### Statistical analysis

Performance metrics, sensitivity, specificity, positive predictive value (PPV), negative predictive value (NPV), and accuracy, were calculated with 95% confidence intervals using the Wilson score method. Cohen’s kappa coefficient was utilized to evaluate the concordance between the Bionote MERS-CoV antigen test and MERS-CoV RT-qPCR results. The ROC curve analysis was performed to determine LOD thresholds.

ANOVA was employed to compare MERS-CoV isolates from clade A (accession: NC_019843.3) and clade C (accession: OR742171.1) and evaluate differences in detection thresholds. Statistical analyses were performed using R statistical software version 4.3.0, following the STARD 2015 guidelines for diagnostic accuracy studies (21). This study adheres to the World Organisation for Animal Health (WOAH) manual of diagnostic tests and vaccines for terrestrial animals validation guidelines (20).

## Results

### Assay performance test for MERS-CoV clade C strains

**Figure 1:**
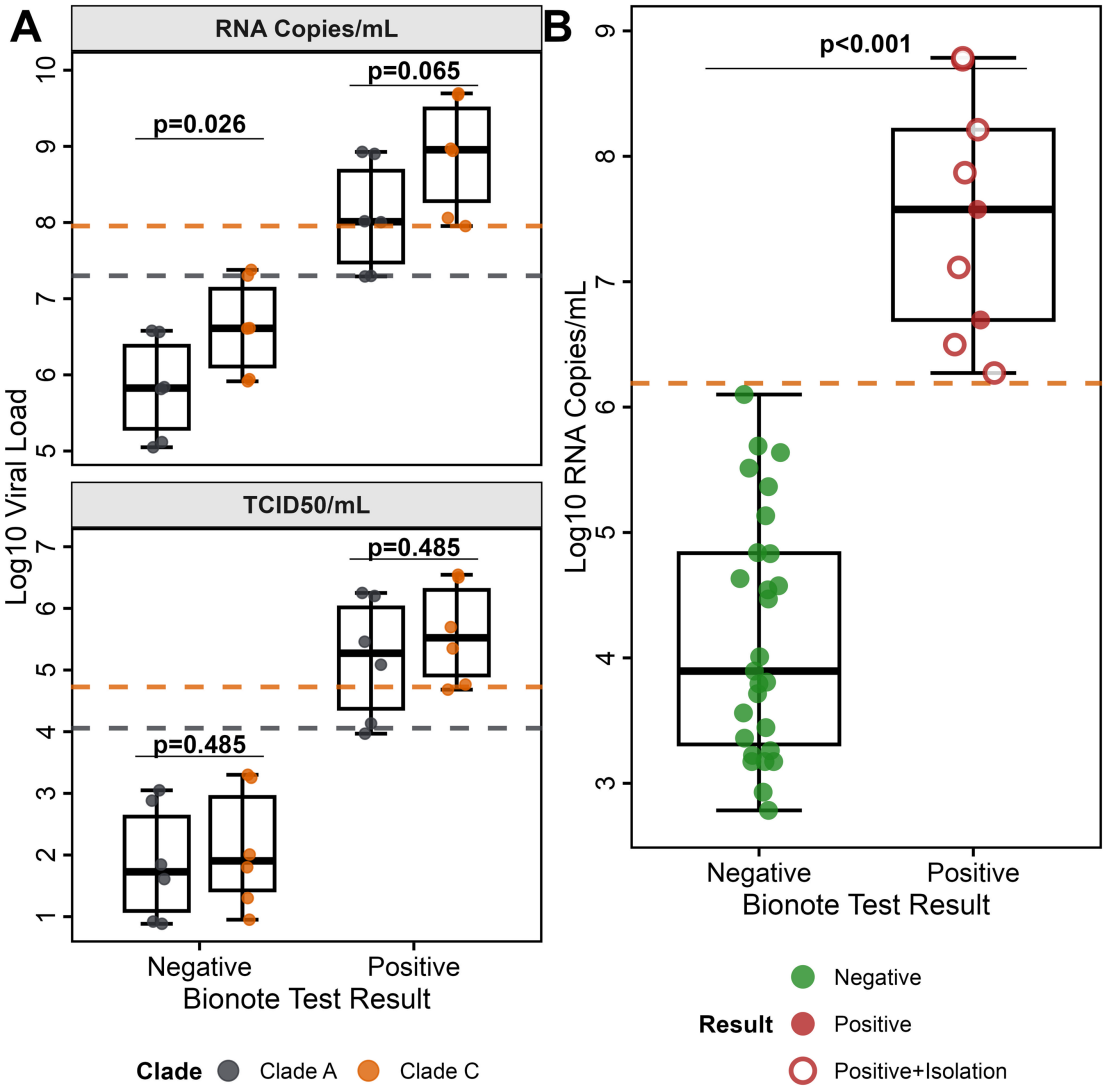
Bionote MERS-CoV antigen test kit clade comparison and validation analysis. **(A)** MERS-CoV clade performance comparison: Detection sensitivity for MERS-CoV clade A (EMC/2012, gray) and clade C (Kenya/9954, orange) using serial dilutions (10^6^ to 10^1^) of MERS-CoV RNA genome copies. The upper panel shows RNA genome copies/ml; the lower panel shows TCID_50_/ml. Box plots display median, quartiles, and range for each test result category by clade. For RNA copies/ml, the LOD for clade A is 2.0 × 10^7^ RNA copies/ml (gray dashed line) and clade C is 9.0 × 10^7^ RNA copies/ml (orange dashed line). For TCID_50_/ml, the LOD for clade A is 1.14 × 10^4^ TCID_50_/ml (gray dashed line) and clade C is 5.32 × 10^4^ TCID_50_/ml (orange dashed line). **(B)** Validation study: Bionote MERS-CoV antigen test performance using pre-tested MERS-CoV RT-qPCR-positive field samples (*n* = 36). Green circles represent MERS-CoV antigen test-negative samples, red filled circles represent antigen test-positive samples without successful viral isolation, and red circles with white centers represent antigen test-positive samples with successful virus isolation (*n* = 7). All samples containing infectious MERS-CoV were tested antigen-positive. Antigen detection sensitivity was 100% for samples above LOD (≥1.3 × 10^6^ copies/ml) (orange dashed line).

The commercial Bionote MERS-CoV rapid antigen test is based on the N protein of MERS-CoV EMC, clade A, and has been validated with MERS-CoV clade A/B samples (14). Camels on the African continent harbor phylogenetically distinct MERS-CoV clade C strains that display considerable sequence heterogeneity, with the N protein containing approximately 20 polymorphic sites relative to the prototypic EMC/2012 isolate (Supplementary Figure 2). The EMC N-specific peptide utilized to generate the anti-N monoclonal antibody (mAb) in this assay (14) contains an amino acid polymorphism at position 178 (L178V) that is conserved across all clade C variants (Supplementary Figure 2). To rule out that the N-specific polymorphism affects the assay performance, a MERS-CoV clade-specific evaluation was conducted using representative clade A (accession: NC_019843.3) and clade C (accession: OR742171.1) isolates. We prepared and tested serial dilutions of Vivaspin-concentrated virus stocks from 10^6^ to 10^1^ based on TCID_50_/ml titers. The data for the respective dilutions were clustered in groups according to a negative and a positive rapid antigen test outcome (Figure 1A). To determine the respective LOD in viral RNA copies/ml and infectious units (TCID_50_/ml), all applied virus dilutions were quantified by RT-qPCR (upE assay) and re-titrated by a TCID_50_-based assay. Viral load analysis revealed comparable LODs for both MERS-CoV variants: For RNA copies/ml, clade A had an LOD of 2.0 × 10^7^ RNA copies/ml (Figure 1A, upper panel, dashed gray line) compared to clade C with 9.0 × 10^7^ RNA copies/ml (Figure 1A, upper panel, dashed orange line). In the case of TCID_50_/ml (Figure 1A, lower panel, dashed gray) determination, clade A had an LOD of 1.14 × 10^4^ TCID_50_/ml whereas clade C had an LOD of 5.32 × 10^4^ TCID_50_/ml (Figure 1A, lower panel, dashed orange line). For all antigen-positive samples, we did not observe statistically significant differences between detection thresholds (RT-qPCR, *p* = 0.065, TCID_50_, *p* = 0.485; Mann–Whitney U test; Figure 1A). Within the negative-antigen test groups, the TCID_50_ was highly comparable whereas the viral RNA copies/ml showed a significant difference. Based on RNA copies per ml, the coefficient of variation between clades was 11.7%, indicating moderate variability in detection performance. All observed LODs were highly comparable to commonly applied N-based SARS-CoV-2 rapid antigen tests (22, 23).

### Diagnostic performance metrics analysis for camel samples containing MERS-CoV clade C

#### Performance metrics evaluation

An in-depth MERS-CoV rapid antigen test performance evaluation was conducted using 2,736 archived, MERS-CoV clade C RT-qPCR-pretested, camel nasal swab samples previously collected during our MERS-CoV surveillance study in Kenya (7). The sample set included 36 MERS-CoV RNA-positive samples (1.32, 95% CI, 0.95–1.80%). The viral RNA concentrations in the 36 MERS-CoV RNA-positive camel samples ranged from 6.08 × 10^3^ RNA copies/ml to 6.1 × 10^8^ RNA copies/ml (Figure 1B).

For the applied field sample set, the Bionote MERS-CoV antigen test achieved a specificity of 100.0% (95% CI, 99.9–100.0%), with all 2,700 MERS-CoV RNA-negative samples accurately identified as negative, resulting in no false positives (summary in Table 1). The rapid antigen test identified 9 out of 36 MERS-CoV RNA-positive samples as true positives resulting in a diagnostic sensitivity of 25%. All 9 MERS-CoV antigen-positive samples had viral RNA concentrations above an LOD of 1.3 × 10^6^ RNA copies/ml.

**Table 1:** Comprehensive performance evaluation of the Bionote MERS-CoV antigen test kit.

Metric	Value
^1^Archived camel nasal samples characteristics	
Total samples tested	2,736
RT-qPCR-positive samples	36
RT-qPCR positivity rate (%)	1.3
Diagnostic performance metrics	
Sensitivity (based on the complete set of archived samples) (%)	25.0 (12.1–42.2)
Sensitivity (based on samples above the LOD of 10^6^) (%)	100.0 (99.9–100.0)
Sensitivity (based on samples with successful virus isolation) (%)	100.0 (99.9–100.0)
Specificity (%)	100.0 (99.9–100.0)
PPV (%)	100.0 (66.4–100.0)
NPV (%)	99.0 (98.6–99.3)
Accuracy (%)	99.0 (98.6–99.3)
Cohen’s kappa	0.397 (0.008–0.046)
Positive likelihood ratio	∞
Negative likelihood ratio	0.75
Viral load thresholds	
Area under curve (ROC)	1.0 (1.0–1.0)
Optimal viral load threshold (log₁₀)	6.19
Optimal viral load threshold (copies/ml)	1.53 × 10^6^
95% detection probability threshold (copies/ml)	1.58 × 10^6^
Estimated LOD (copies/ml)	5.01 × 10^5^
LOD (%)	90.0 (55.5–99.7)
Error analysis	
False negative samples (complete sample set)	27
Median viral load of false negatives (copies/ml)	7.85 × 10^3^
False positive samples	0
False positive rate (%)	0.00

PPV, Positive Predictive Value; NPV, Negative Predictive Value; LOD, Limit of Detection. ^1^Data from camel nasal swabs collected in Kenya 2022–2023. ^2^All confidence intervals are 95% CI calculated using exact binomial method where applicable.

To explore the connection of antigen test-positivity and infectiousness, we tested a subset of 20/36 MERS-CoV RT-qPCR-positive camel nasal samples (threshold >10^4^ RNA copies/ml) in virus isolation attempts. In 7/20 samples we retrieved MERS-CoV isolates (Figure 1B, open circles). The 7 virus isolates contained RNA concentrations above the LOD range of 2 − 9 × 10^7^ RNA copies/ml. All 7 samples had also been tested positive in the MERS-CoV rapid antigen test suggesting a good correlation between MERS-CoV antigen positivity and infectiousness (Figure 1B, Supplementary Table 1).

Twenty-seven out of 36 MERS-CoV RNA-positive samples with low viral RNA concentrations below the determined LOD were tested negative in the Bionote test (Table 1). Based on the complete sample set (*n* = 2,736), the rapid antigen test had a moderate sensitivity of 25.0% (95% CI, 16.2–36.1%). However, when taking into account only MERS-CoV RNA-positive samples above the LOD, the rapid antigen test had a sensitivity of 100% (95% CI, 99.9–100.0%). The rapid antigen test yielded an infinite positive likelihood ratio and a negative likelihood ratio of 0.75 (95% CI, 0.64–0.84), due to no detection of false positives.

As summarized in Table 1, the predictive value analysis indicated a PPV of 100.0% (95% CI: 66.4–100.0%). The NPV was 99.0% (95% CI: 98.7–99.3%), indicating a significant ratio of true negatives to false negatives within this low-prevalence population. The overall diagnostic accuracy was 99.0% (95% CI: 98.7–99.3%), largely attributed to the large number of true negative results within the negative sample population. The agreement analysis between the Bionote MERS-CoV antigen test and MERS-CoV RT-qPCR results using Cohen’s Kappa coefficient had a value of *κ* = 0.40 (95% CI: 0.23–0.56), signifying fair agreement.

#### Viral load threshold determination

**Figure 2:**
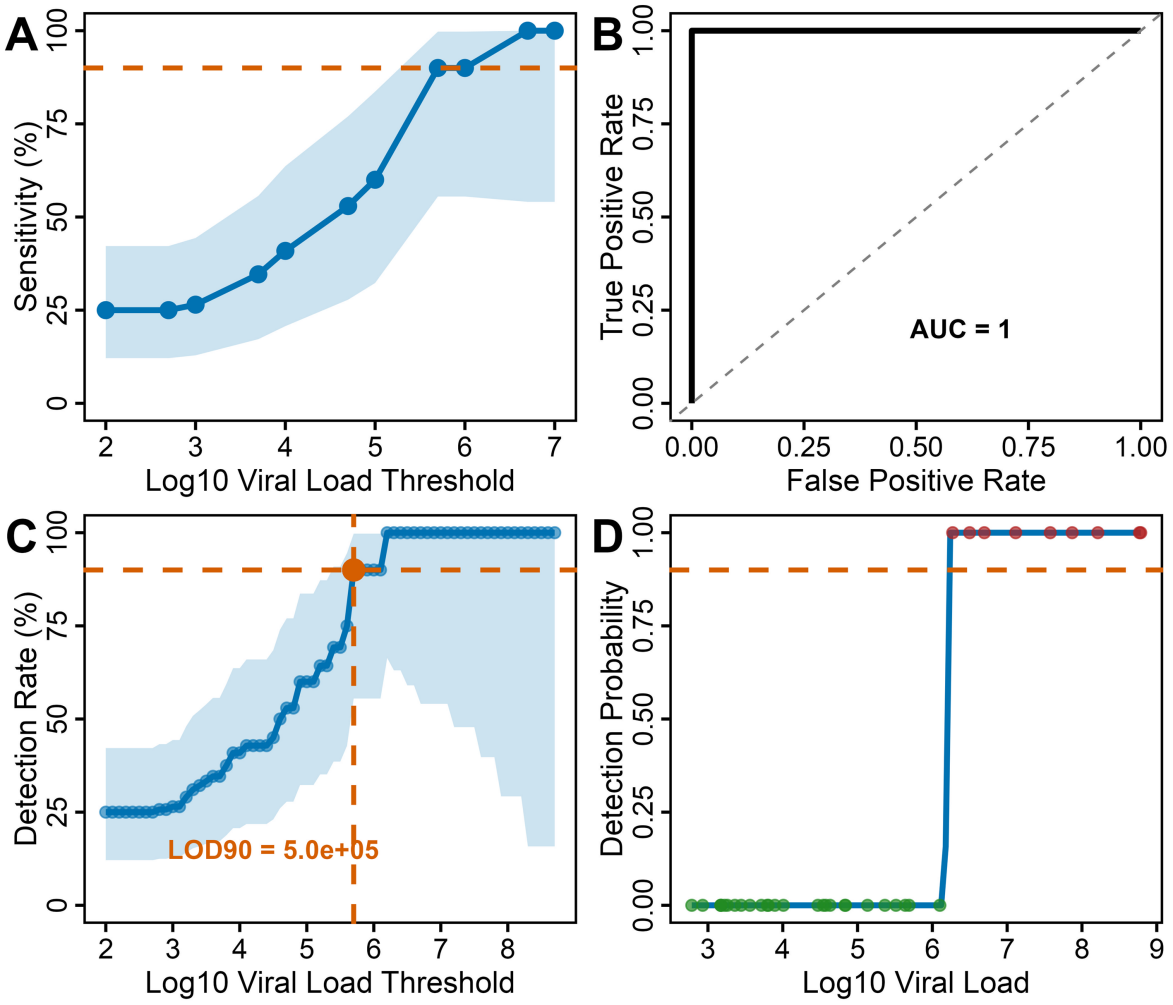
Threshold and detection analysis for the Bionote MERS-CoV rapid antigen test: **(A)** Sensitivity threshold analysis: Test sensitivity across different viral load ranges with confidence intervals. Performance exceeds the 95% sensitivity threshold at high viral loads (>10^6^ copies/ml) **(B)** ROC curve analysis: ROC curve analysis of MERS-CoV viral copies/ml as a predictor of the Bionote MERS-CoV antigen test results among the MERS-CoV RT-qPCR-positive samples (*n* = 36). Excellent discrimination (AUC = 1.0) was determined by Youden’s J statistic. The diagonal dashed line represents random classification (AUC = 0.5). **(C)** Detection rate by viral load threshold: Sensitivity progressively increased with viral load, reaching 90% detection at the estimated modeled LOD of 5.01 × 10^5^ copies/ml. Blue shading represents 95% confidence intervals. **(D)** Detection probability model: Logistic regression analysis showing a sharp transition from 0% − 100 detection probability around 10^6^ copies/ml, with clear 50% detection threshold.

A viral load threshold analysis revealed a clear bimodal distribution with distinct separation between detected and missed MERS-CoV cases. All 9 Bionote MERS-CoV test-positive samples had viral loads exceeding 10^6^ copies/ml (1.2 × 10^6^ to 8.5 × 10^7^) (Figure 1B), while false negative samples showed significantly lower median viral loads of 7.85 × 10^3^ copies/ml (IQR: 2.1 × 10^3^–4.2 × 10^4^; *p* < 0.0001, Mann–Whitney U test), representing a > 100-fold difference in viral burden. The sensitivity threshold analysis showed a test sensitivity across different viral load ranges (Figure 2A). The test performance exceeded the 95% sensitivity threshold at high viral loads (>10^6^ copies/ml, Figure 2A). ROC curve analysis demonstrated excellent discrimination between detected and missed cases (AUC = 1.0; 95% CI: 1.0–1.0), (Figure 2B) showing a complete separation of viral load distributions. The analysis identified an optimal LOD of 6.19 log₁₀ copies/ml (1.53 × 10^6^ copies/ml) using Youden’s J statistic. Systematic threshold analysis established the LOD for 90% detection probability (LOD_90_) at 5.01 × 10^5^ copies/ml (Figure 2C). Logistic regression modeling confirmed the relationship between MERS-CoV viral load and detection probability (*p* < 0.001), demonstrating a sharp transition zone around 10^6^ RNA copies/ml where detection probability approaches certainty (Figure 2D). At viral loads ≥10^6^ copies/ml, sensitivity reached 100% (9/9 samples), while sensitivity decreased to 0% (0/27 samples) below this threshold.

### Detection of acute MERS-CoV infections in a slaughterhouse

In our previous study (7), we showed a biphasic seasonality of MERS-CoV incidence in a camel slaughterhouse in Isiolo, northern Kenya. We identified two peak periods of MERS-CoV RNA-positive camels in September–November 2022 and February–March 2023. We therefore chose March–April 2024 for a prospective MERS-CoV surveillance study at the Isiolo County camel slaughterhouse. During this period, camels were tested daily (5 days per week) before being slaughtered. In total, 386 camels (approx. 8–12 camels per work day) were tested in close proximity to the slaughterhouse (Supplementary Figure 3). We identified three MERS-CoV antigen-positive camels among 386 tested over the eight-week study period, yielding a prevalence of 0.78% (95% CI: 0.16–2.27%). The three MERS-CoV-antigen positive camels were detected on different days (11^th^, 12^th^ and 23^rd^ March 2024) of the surveillance period, showing the utility of the test in real-time identification of MERS-CoV-infected camels. All three MERS-CoV-antigen test-positive results were confirmed by upE MERS-CoV RT-qPCR, with additional orf1a confirmation showing 100% concordance between the MERS-CoV antigen test and MERS-CoV RT-qPCR results. Viral load quantification revealed viral RNA concentrations of 1.1 × 10^9^, 2.4 × 10^8^, and 8.5 × 10^7^ RNA copies/ml, respectively. All values substantially exceeded the established LOD threshold of 1.53 × 10^6^ copies/ml.

## Discussion

Our in-depth evaluation and field application of the Bionote MERS-CoV antigen test kit resulted in three key findings. First, the test’s detection performance remained consistent across different MERS-CoV clades (including clade C), indicating that it can be reliably used with different MERS-CoV variants. Second, our analysis of 2,736 archived camel nasal samples revealed an experimental LOD of 1.53 × 10^6^ RNA copies/ml and a specificity of 100%. The antigen assay sensitivity was 25% when compared to highly sensitive RT-qPCR but 100% for infectious samples. Third, our prospective slaughterhouse MERS-CoV surveillance identified three MERS-CoV RNA-positive camels using the Bionote MERS-CoV antigen test kit, validating its use for real-time, on-site detection of MERS-CoV outbreaks in camel herds in remote areas.

The Bionote MERS-CoV rapid antigen test was originally established for the detection of MERS-CoV clade A and was validated using camel samples infected with MERS-CoV clade B (14). The comparable experimental LODs (2.0 × 10^7^ vs. 9.0 × 10^7^ RNA copies/ml) for the purified MERS-CoV clade A and C virus isolates or for the MERS-CoV clade C field samples (LOD = 1.53 × 10^6^ RNA copies/ml) demonstrated that the MERS-CoV N protein detection in the rapid antigen test remains sufficiently sensitive for MERS-CoV clade C circulating on the African continent (24). The N epitopes recognized by the monoclonal antibodies are still sufficiently conserved across MERS-CoV clades; however, our analysis revealed at least 20 polymorphisms in the N protein distributed among MERS-CoV clade C strains. The ongoing MERS-CoV evolution (25, 26) emphasizes the necessity of continuously monitoring changes in diagnostic targets. In the comparison of TCID_50_/ml and RNA copies/ml between clade A and clade C, all antigen-positive samples showed no statistically significant differences in detection thresholds. Conversely, within the negative-antigen test groups, TCID_50_ values were highly comparable, while viral RNA copies/ml showed a significant difference. This might be explained by the presence of more defective viral particles in the concentrated MERS-CoV clade C virus stock.

Our experimental performance evaluation of the Bionote MERS-CoV antigen test kit was based on the complete sample set of archived camel nasal samples and revealed that the diagnostic sensitivity depends strongly on the respective reference test. The initial laboratory validation (14) reported 94% sensitivity based on a comparison with TCID_50_ assays. In our assessment, antigen test sensitivity reached 78% based on virus isolation success, and 25% when compared to RT-qPCR. The moderate sensitivity of the rapid antigen test (LOD = 1.53 × 10^6^ RNA copies/ml) in comparison to the highly sensitive RT-qPCR was expected since the LOD of the MERS-CoV-specific upE RT-qPCRs is 291 RNA genome copies/ml (16). Our MERS-CoV RNA-positive sample set (*n* = 36) was conveniently taken from a previous study (7) covering a wide range of viral RNA concentrations, many of them below 10^6^ copies/ml. A cut-off value of 10^5^–10^6^ copies/ml is typical for commercially available viral rapid antigen tests (e.g., SARS-CoV-2, Influenza, RSV) (27, 28). The Bionote antigen test specificity was confirmed to be 100% using a substantial number of samples (>2,700) which is important for MERS-CoV surveillance studies, as it minimizes the possibility of false-positive results. The high specificity makes the test exceptionally useful for targeted surveillance in high-risk transmission areas. This is particularly important for surveillance programs in low- and middle-income countries that prioritize resource allocation toward the highest-risk scenarios rather than comprehensive case identification. In resource-constrained settings, surveillance strategies often rely on high-specificity tests to ensure that positive results reliably indicate transmissible infections. The test’s ability to provide definitive positive results makes it well-suited for surveillance at critical and remote settings such as camel markets, slaughterhouses, and border crossings where spillover risk to humans is elevated.

Similar to the Bionote MERS-CoV antigen test kit performance metrics, the modeled MERS-CoV LOD_90_ had a threshold of 5 × 10^5^ RNA copies/ml, with excellent discrimination between detected and missed cases (AUC = 1.0). The four-log separation between detected cases (>10^6^ copies/ml) and false negatives (median 7.85 × 10^3^ copies/ml) shows that the test kit selectively identifies MERS-CoV infections in camels with high viral loads. Previous SARS-CoV-2 studies showed that only samples with virus RNA concentrations above 10^5^–10^6^ RNA copies/ml are rapid antigen test-positive and contain infectious particles (9). In addition, systematic reviews on coronavirus infectivity have demonstrated that viral loads above 10^6^ copies/ml strongly correlate with culturable virus and transmission potential (29) suggesting that our viral load threshold corresponds with infectious virus concentrations. This is particularly relevant for MERS-CoV zoonotic transmission, where spillover events at the camel-human interface typically involve intensive exposure scenarios during peak viral shedding phases (30). Noteworthy, the time point of sampling is critical as viral RNA detection, antigen positivity, and viral culturability experience different dynamics (31). Whereas during the peak of infection, high viral load, antigen positivity, and infectiousness often match, the possibility for virus isolation wanes much faster than RNA or antigen detection (31). Despite this, MERS-CoV antigen tests seem to be very useful for the identification of camels with the highest transmission potential, enabling resource-efficient surveillance focused on camels posing the greatest zoonotic risk.

The prospective slaughterhouse surveillance demonstrated the successful translation of the kit’s laboratory evaluation findings to field implementation. We confirmed that the three MERS-CoV antigen-positive camels had consistently high viral loads (>10^7^ RNA copies/ml) and were, based on this study, most likely infectious. For technical reasons, we were unfortunately not able to attempt virus isolation trials yet. Based on previous studies, we can postulate that we sampled the three camels during or after days 7–10 post infection (32), which constitutes the peak phase of infection, allowing the possibility that transmission might have occured in the slaughterhouse holding pens (7). Our finding has direct occupational health significance, as studies in Kenya documented MERS-CoV seropositivity among slaughterhouse workers, with specific risk factors (5, 7). Although highly sensitive and specific qPCRs are favorably applied for diagnostics, the deployment of the Bionote MERS-CoV antigen test kit at camel slaughterhouses addresses an occupational health gap where rapid case identification can aid the implementation of biosafety protocols.

This study has some limitations that should be considered when interpreting the results. First, the moderate overall diagnostic sensitivity of 25% compared to RT-qPCR may limit the test’s utility for comprehensive surveillance programs where detection of all infected animals is required. This sensitivity must be interpreted in the context of the test’s intended purpose for identifying infectious cases rather than all RNA-positive animals. Second, the validation was performed using archived samples that may have undergone freeze–thaw cycles, potentially affecting antigen stability and test performance compared to freshly taken samples. Finally, inter-operator variability assessment was limited to a single central abattoir, and broader multi-site validation would strengthen confidence in field performance across diverse settings. Despite these limitations, the test’s high specificity and ability to identify infectious cases makes it valuable for targeted surveillance in high-risk settings where rapid results can inform immediate public health action.

## Conclusion

The Bionote MERS-CoV antigen test kit enables targeted detection at high-risk human–camel interfaces where zoonotic transmission is most likely. Our prospective slaughterhouse surveillance demonstrates the practical integration of rapid testing into operational surveillance systems, validating real-time outbreak detection capabilities. The sensitivity of the antigen test was comparable to other commonly applied rapid tests with an LOD of 10^6^ viral RNA copies/ml which typically correlates with the threshold of infectiousness for respiratory viruses. The high specificity ensures reliable positive identification without causing false alarms in resource-limited settings. This approach prioritizes identification of transmission-relevant cases over exhaustive screening, providing a cost-effective framework for MERS-CoV surveillance that balances epidemiological significance with operational feasibility in challenging field environments.

## Data Availability

The original contributions presented in the study are included in the article/Supplementary material, further inquiries can be directed to the corresponding authors.
