# Supplementary material for: On-site detection of MERS-CoV infections in a camel slaughterhouse in Kenya using a commercial rapid antigen test
Source: Front Vet Sci. 2025 Sep 15;12:1675847. doi: 10.3389/fvets.2025.1675847 (PMC12477690; doi:10.3389/fvets.2025.1675847)
Supplement: Supplementary file 1 [file Data_Sheet_1.pdf]

Supplementary Material

Supplementary Figure 1

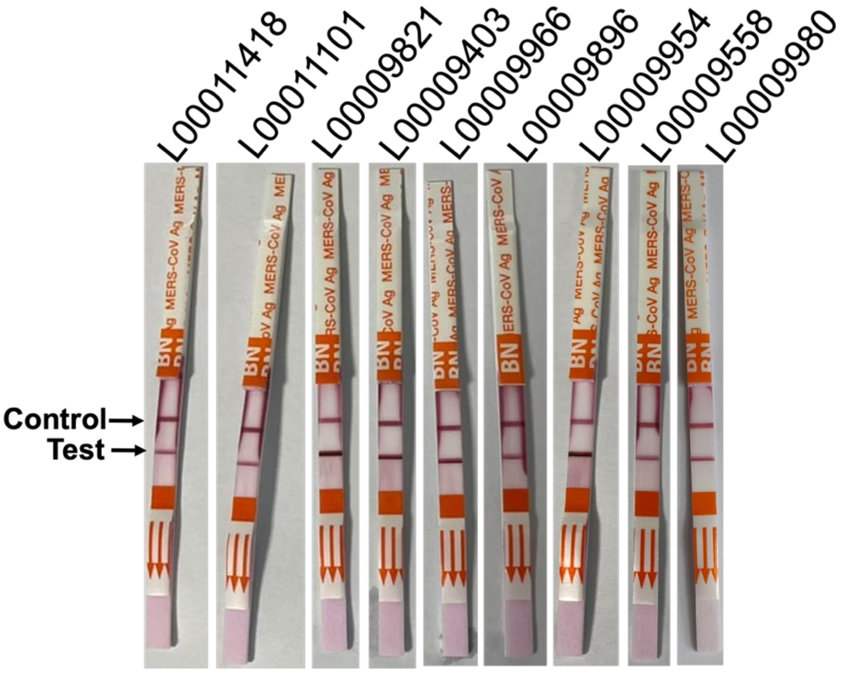

Supplementary Figure 1: MERS-CoV rapid antigen test results. Results of the 9 positive Bionote MERS-CoV rapid antigen tests performed on archived nasal swab samples from dromedary camels. The control line (C) and test line (T) positions. A positive result is indicated by the presence of both control and test lines, while a negative result shows only the control line.

Supplementary Figure 2

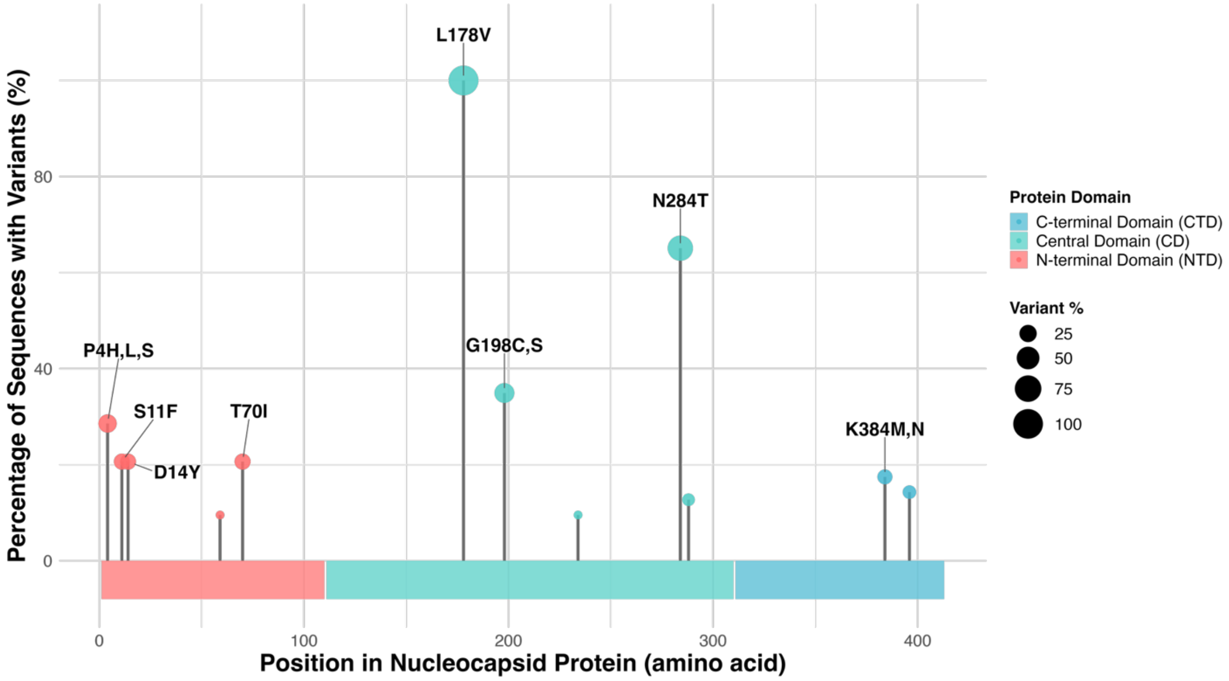

Supplementary Figure 2: MERS-CoV nucleocapsid protein polymorphisms: Amino acid variants across the MERS-CoV Clade C nucleocapsid protein (n=63 sequences) compared to Clade A EMC/2012 reference. Variants are shown for positions occurring in ≥15% of sequences, with labels displayed for those present in ≥20% of sequences. Protein domains: N-terminal (red), central (teal), C-terminal (blue). Circle size indicates variant frequency. Notable polymorphisms include L178V and N284T in functional domains.

Supplementary Figure 3

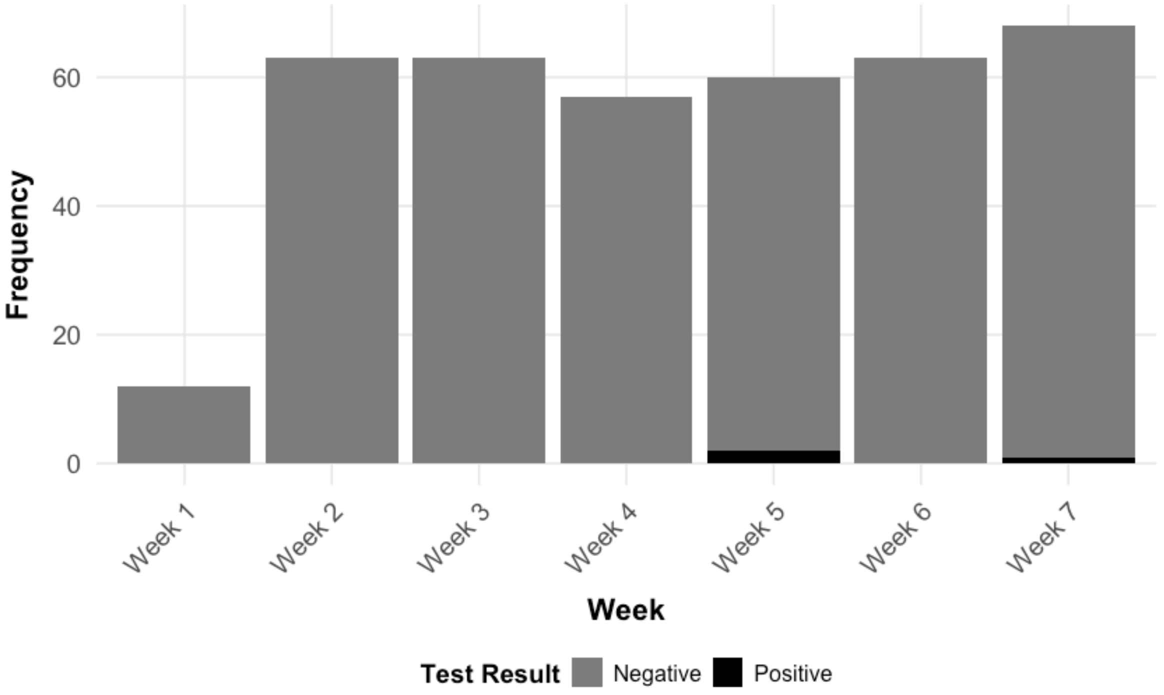

Supplementary Figure 3: MERS-CoV antigen detection using the Bionote antigen test in Isiolo abattoir study, March 2024- April 2024. Weekly distribution of Bionote MERS-CoV rapid antigen test results from abattoir surveillance over 13 weeks. Total samples tested n=386. Overall MERS-CoV antigen positivity rate was 0.78% (3 positive camel samples). Black bars represent negative results; gray bars represent positive results.

Supplementary Table 1

Supplementary Table 1: MERS-CoV virus isolation attempts on Caco-2 cells

Animal ID	Concentration [copies/mL]	Bionote test	Viral isolation result
**L00009821**	$1.63x10^8$	Positive	Positive
**L00009820**	$6.91x10^4$	Negative	Negative
**L00009403**	$1.87x10^6$	Positive	Positive
**L00009558**	$1.30x10^7$	Positive	Positive
**L00009662**	$4.34x10^5$	Negative	Negative
**L00009954**	$3.15x10^6$	Positive	Positive
**L00009980**	$6.79x10^6$	Positive	Positive
**L00009966**	$5.88x10^8$	Positive	Positive
**L00009896**	$2.35x10^8$	Positive	Positive
**L00009913**	$2.32x10^5$	Negative	Negative
**L00009817**	$4.89x10^5$	Negative	Negative
**L00011101**	$3.77x10^7$	Positive	Negative
**L00011081**	$2.95x10^4$	Negative	Negative
**L00011418**	$4.95x10^6$	Positive	Negative
**L00011505**	$1.26x10^6$	Negative	Negative
**L00011530**	$3.76x10^4$	Negative	Negative
**L00011542**	$4.29x10^4$	Negative	Negative
**L00011549**	$1.36x10^5$	Negative	Negative
**L00011622**	$3.26x10^5$	Negative	Negative
**L00011675**	$6.78x10^4$	Negative	Negative
